# Supplementary material for: Delineation of signaling routes that underlie differences in macrophage phenotypic states
Source: NAR Mol Med. 2025 Apr 25;2(2):ugaf013. doi: 10.1093/narmme/ugaf013 (PMC12430007; doi:10.1093/narmme/ugaf013)
Supplement: ugaf013_Supplemental_Files [file ugaf013_Supplemental_Files.zip › Supplementary_Figures.pdf]

Supplementary Figures

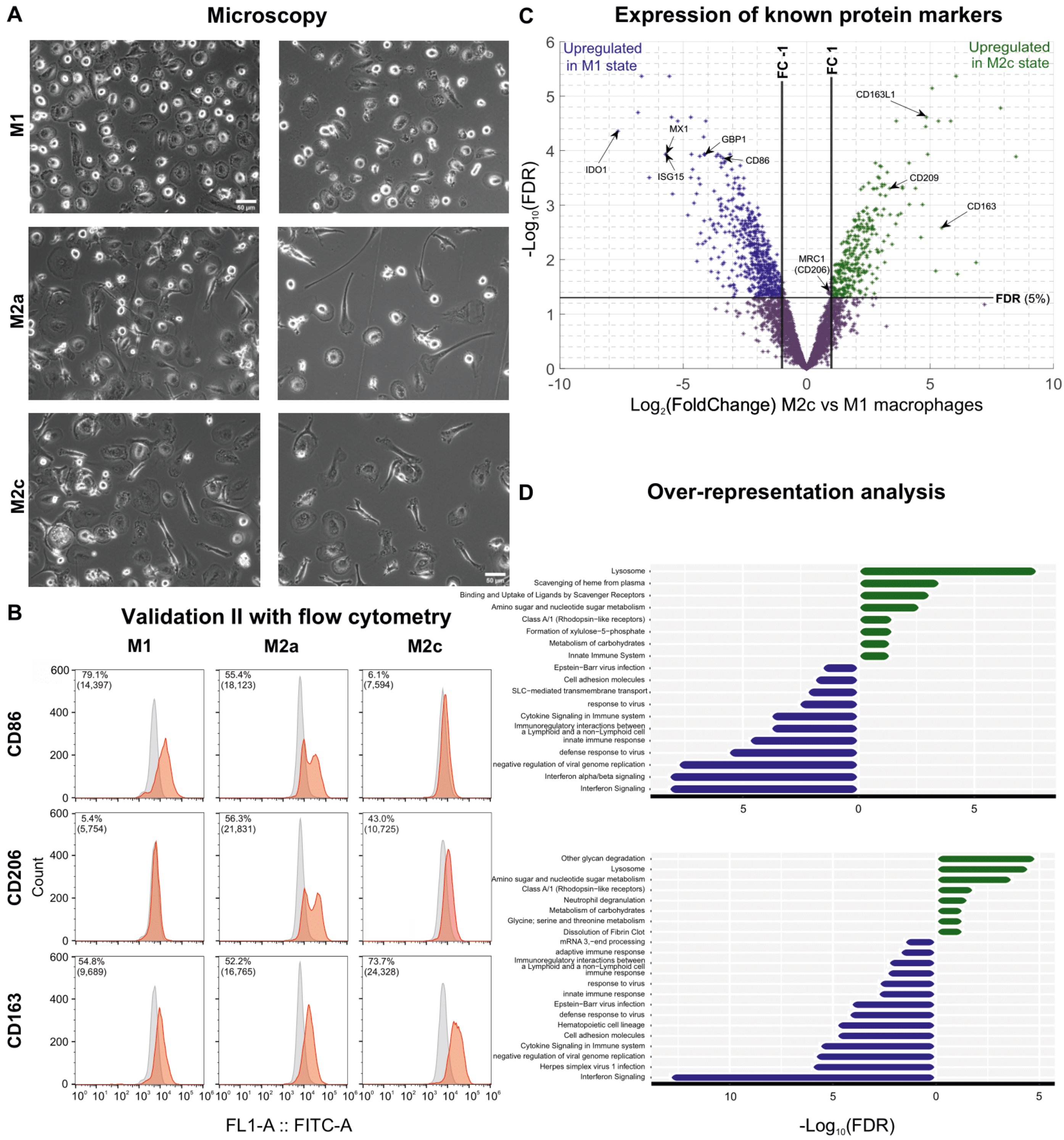

Figure S1. Morphological and molecular properties of primary *in vitro* polarized M1, M2a and M2c phenotypic states. (**A**) M1, M2a and M2c macrophages were differentiated and polarized from blood-derived monocytes isolated from buffy coats. Shown are images taken after 6 days of differentiation (M1: GM-CSF, M2a/M2c: M-CSF) and 2 days of polarization (M1: LPS/IFN-γ, M2a: IL-4/IL-13, M2c: IL-10). The images were taken from two donors with a Primovert microscope (Carl Zeiss). (**B**) Flow cytometry profiles (Gallios/Beckman Coulter) for the polarization markers CD86, CD206 and CD163 of *in vitro* polarized macrophages phenotypes in the second of the two tested donors with antigens of interest shown in red and negative or isotype controls shown in grey. Percentages of positive cells, together with the

median fluorescence intensity in brackets, are indicated on each individual plot. Y-axes are truncated. (**C**) Volcano plot illustrating protein marker expressions in terms of Log2FC values on the X axis against -Log_{10}(FDR) values on the Y axis in a comparison of the M1 and M2c phenotypic states. Differentially expressed proteins are colored blue and green, depending on the directionality of the expression change. (**D**) Significant (FDR < 0.05) over-representation of differentially expressed proteins (Log_2FC ≥ 1, FDR < 0.05) between M1 compared to M2a (top) and M2c (bottom) macrophages within KEGG and Reactome pathways. Blue represents significant pathways related to the M1 phenotypic state, while M2a and M2c are represented in green.

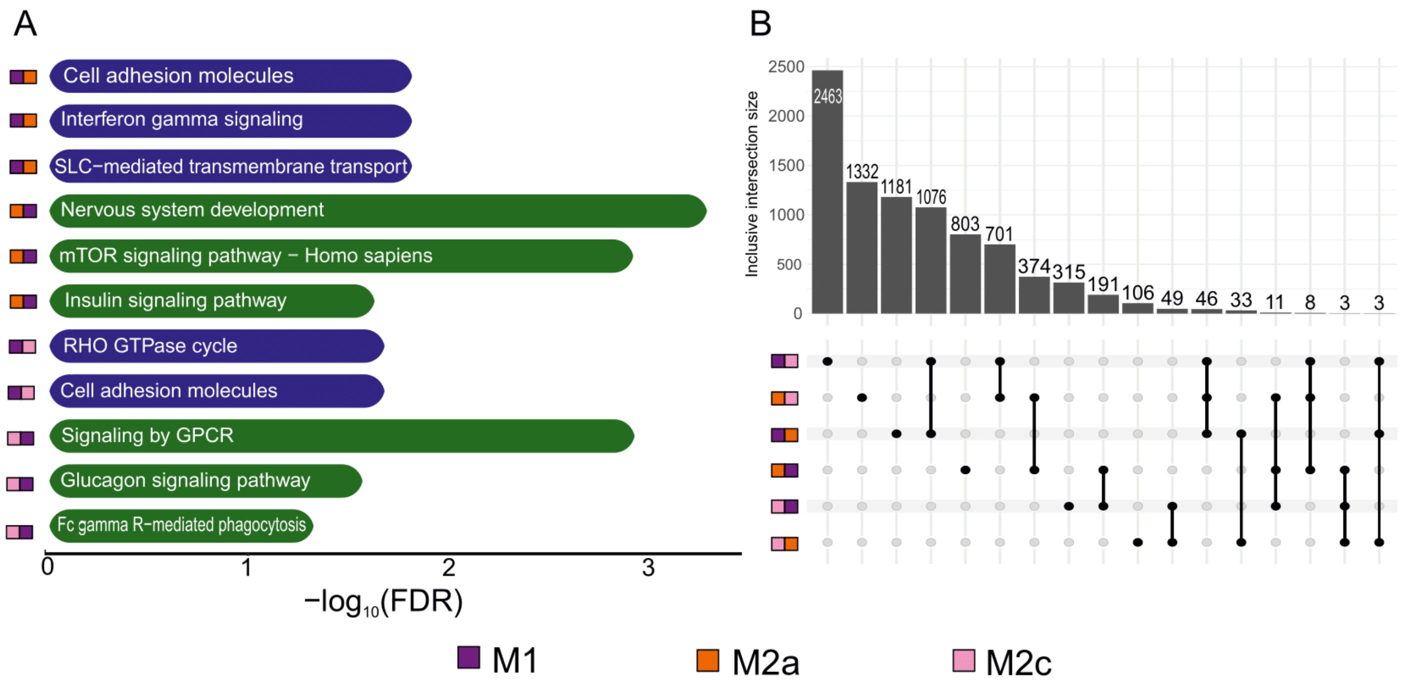

Figure S2. Analysis of peptides with significantly different phosphorylation levels in the studied conditions. (**A**) Significant (FDR < 0.05) over-representation of proteins with differentially regulated phosphoresidues (Log2FC ≥ 1, FDR < 0.05) between M1 compared to M2 macrophages within KEGG and Reactome pathways. Blue represents significant pathways related to the M1 phenotypic state, while M2a and M2c are represented in green. (**B**) UpSet plot representation summarizing the differentially expressed phosphopeptides between all the studied macrophage phenotypic states with minimum intersection size of three. The two squares indicate the phenotype in which upregulation occurs relative to the other phenotype (the left square represents a condition in which the phosphopeptides were upregulated and the right square a condition with which the comparison was done).

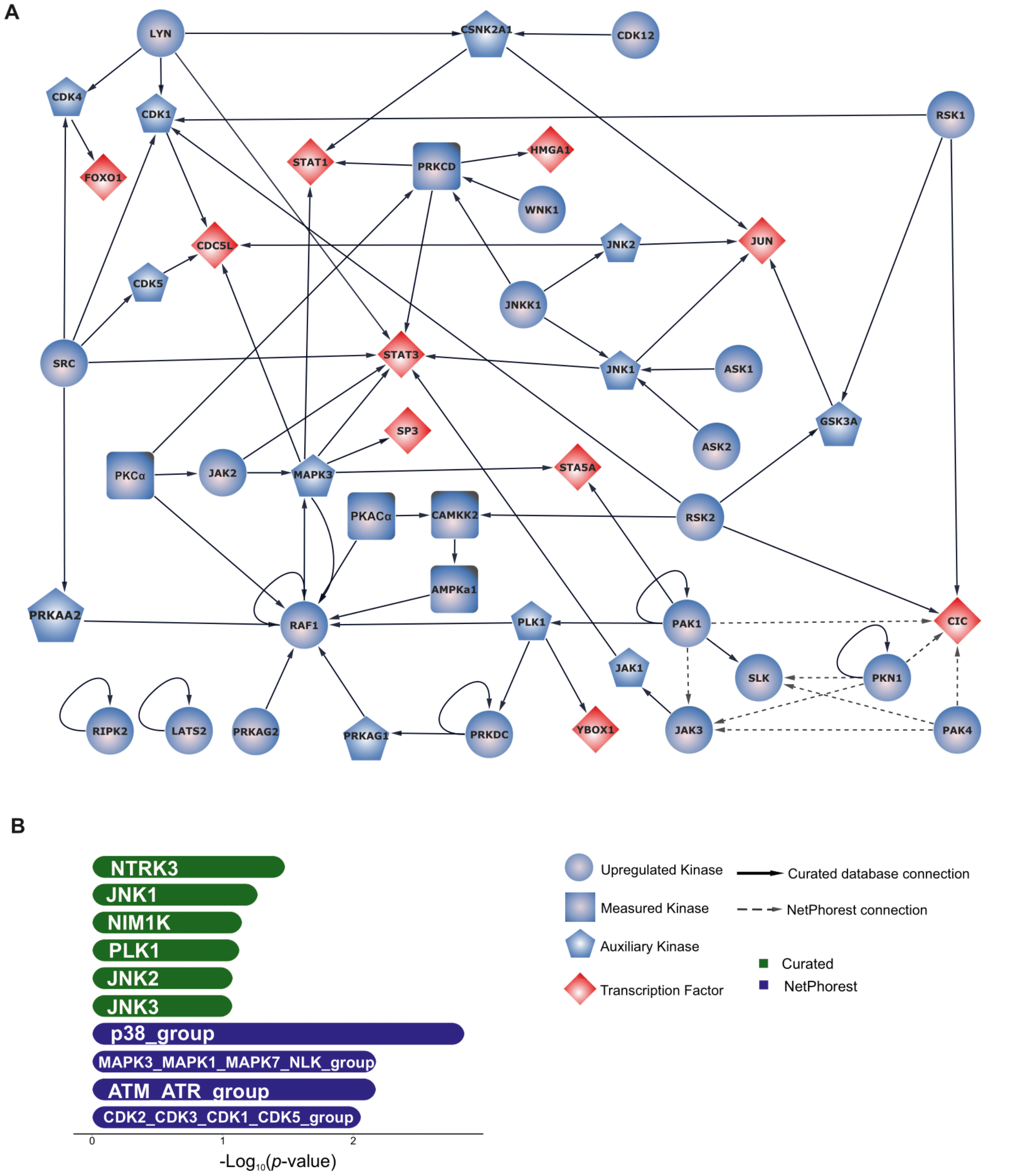

Figure S3. (**A**)Directional kinase-kinase signaling networks centered around the main upregulated kinases of the **M1 compared to M2a** phenotypic state. Within the network, there are modeled kinases with at least one upregulated phosphopeptide (spherical objects) and TFs with upregulated phosphopeptides (rhombic objects). To overcome the challenge of missing values, we allowed the inclusion of known upstream regulatory kinases, which could connect two upregulated kinases or transcription factors in the network, even if they were not measured (pentagon-shaped objects) or they were measured but did not have significantly different levels between the states (square objects). Curated kinase-substrate knowledge from PhosphoSitePlus and four other databases was used to connect the kinases (solid lines) as well as the NetPhorest prediction tool to complement the missing knowledge (dotted lines). A connection edge between two upregulated kinases makes reference to a connection between a kinase that has upregulated phosphorylated

residues and a specific peptide that was found to be upregulated as well. This is also valid for the edges with TFs. If an upregulated kinase is linked to a measured but not upregulated kinase, this means that the residues of the latter were not found to be differentially expressed. The presented kinase-kinase signaling maps highlight the signaling transduction routes as were measured in our study. (**B**) Upstream kinase activity assessment highlighting the top predicted upstream kinases responsible for the phosphorylation of the upregulated phosphopeptides of **M1 compared to M2a** phenotypic state. The analysis was based either on curated phosphorylation databases knowledge or NetPhorest predictions, each analysis relying on a two-sided Fisher's exact *t*-test where the background was represented by all phosphopeptides measured in the respective phenotypes (see Methods).

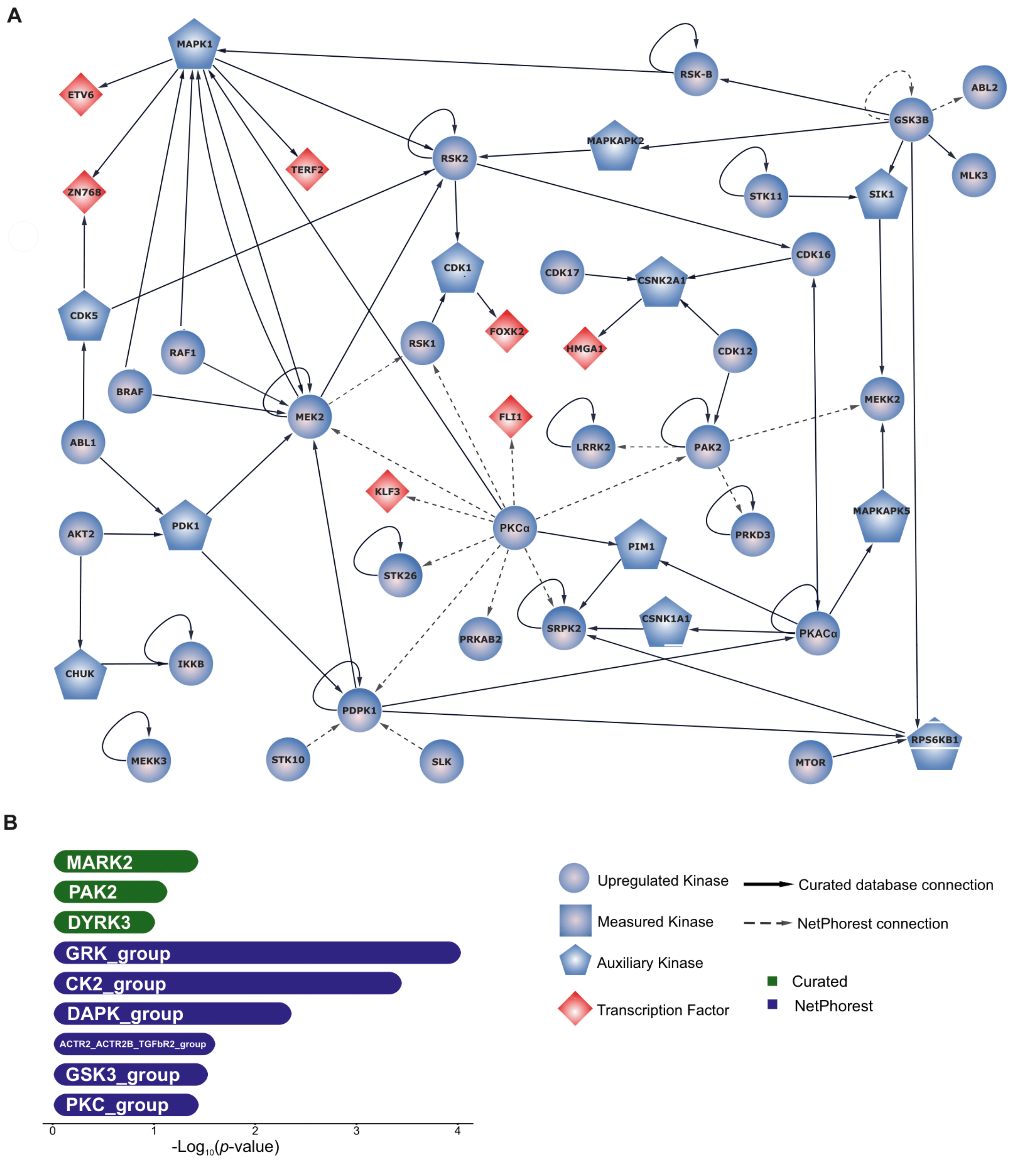

Figure S4. (**A**) Directional kinase-kinase signaling networks centered around the main upregulated kinases of the **M2a compared to M1** phenotypic state. Within the network, there are modeled kinases with at least one upregulated phosphopeptide (spherical objects) and TFs with upregulated phosphopeptides (rhombic objects). To overcome the challenge of missing values, we allowed the inclusion of known upstream regulatory kinases, which could connect two upregulated kinases or transcription factors in the network, even if they were not measured (pentagon-shaped object) or they were measured but did not have significantly different levels between the states (square objects). Curated kinase-substrate knowledge from PhosphoSitePlus and four other databases was used to connect the kinases (solid lines) as well as the NetPhorest prediction tool to complement the missing knowledge (dotted lines). A connection edge between two upregulated kinases makes reference to a connection between a kinase that has upregulated phosphorylated

residues and a specific peptide that was found to be upregulated as well. This is also valid for the edges with TFs. If an upregulated kinase is linked to a measured but not upregulated kinase, this means that the residues of the latter were not found to be differentially expressed. The presented kinase-kinase signaling maps highlight the signaling transduction routes as were measured in our study. (**B**) Upstream kinase activity assessment highlighting the top predicted upstream kinases responsible for the phosphorylation of the upregulated phosphopeptides **M2a compared to M1** phenotypic state. The analysis was based either on curated phosphorylation databases knowledge or NetPhorest predictions, each analysis relying on a two-sided Fisher's exact *t*-test where the background was represented by all phosphopeptides measured in the respective phenotypes (see Methods).

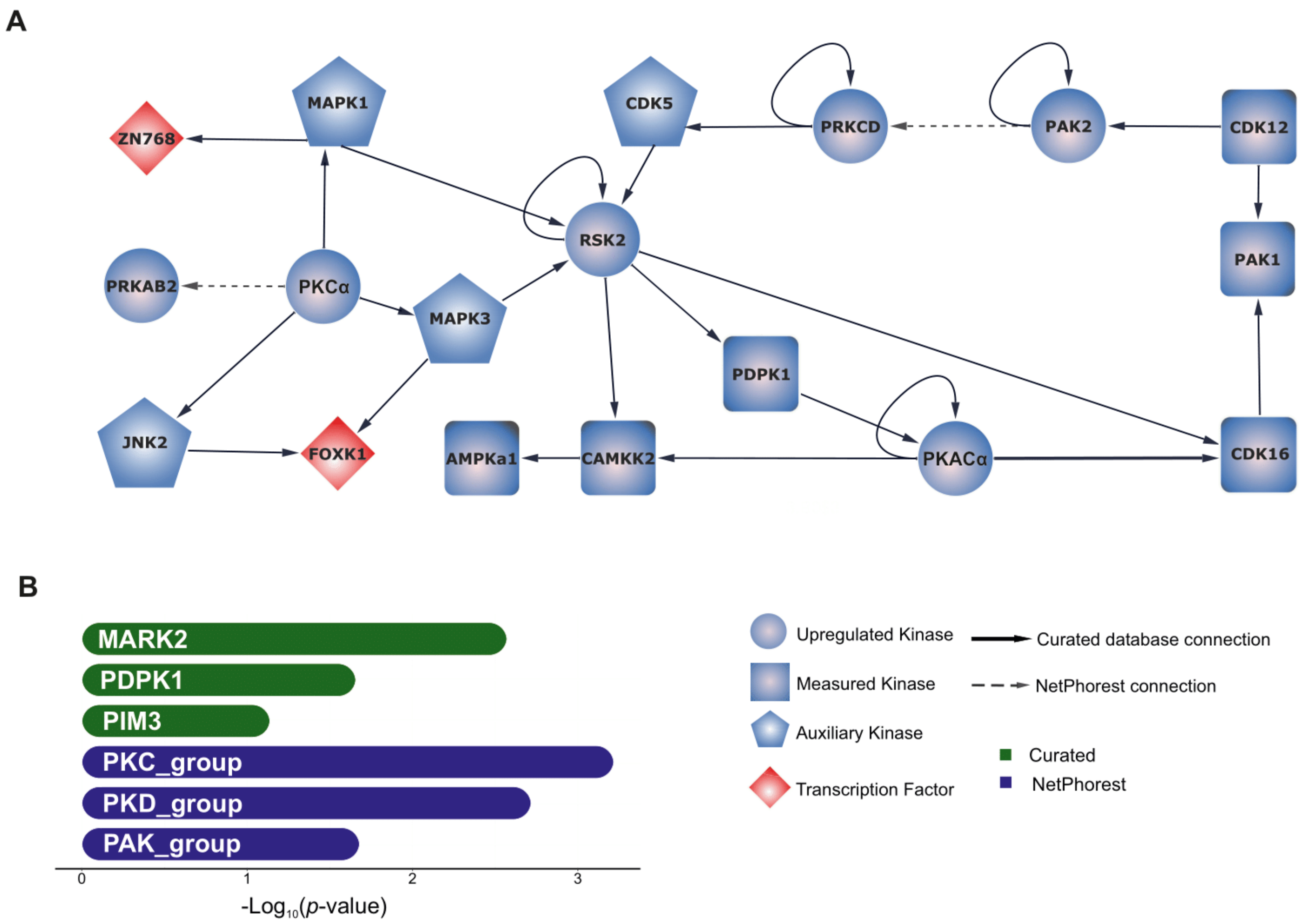

Figure S5. (**A**) Directional kinase-kinase signaling networks centered around the main upregulated kinases of the **M2c compared to M1** phenotypic state. Within the network, there are modeled kinases with at least one upregulated phosphopeptide (spherical objects) and TFs with upregulated phosphopeptides (rhombic objects). To overcome the challenge of missing values, we allowed the inclusion of known upstream regulatory kinases, which could connect two upregulated kinases or transcription factors in the network, even if they were not measured (V-shaped object) or they were measured but did not have significantly different levels between the states (square objects). Curated kinase-substrate knowledge from PhosphoSitePlus and four other databases was used to connect the kinases (solid lines) as well as the NetPhorest prediction tool to complement the missing knowledge (dotted lines). A connection edge between two upregulated kinases makes reference to a connection between a kinase that has upregulated phosphorylated residues and a specific peptide that was found to be upregulated as well. This is also valid for the edges with TFs. If an upregulated kinase is linked to a measured but not upregulated kinase, this means that the residues of the latter were not found to be differentially expressed. The presented kinase-kinase signaling maps highlight the signaling transduction routes as were measured in our study. (**B**) Upstream kinase activity assessment highlighting the top predicted upstream kinases responsible for the phosphorylation of the upregulated phosphopeptides of **M2c compared to M1** phenotypic state. The analysis was based either on curated phosphorylation databases knowledge or NetPhorest predictions, each analysis relying on a two-sided Fisher's exact *t*-test where the background was represented by all phosphopeptides measured in the respective phenotypes (see Methods).

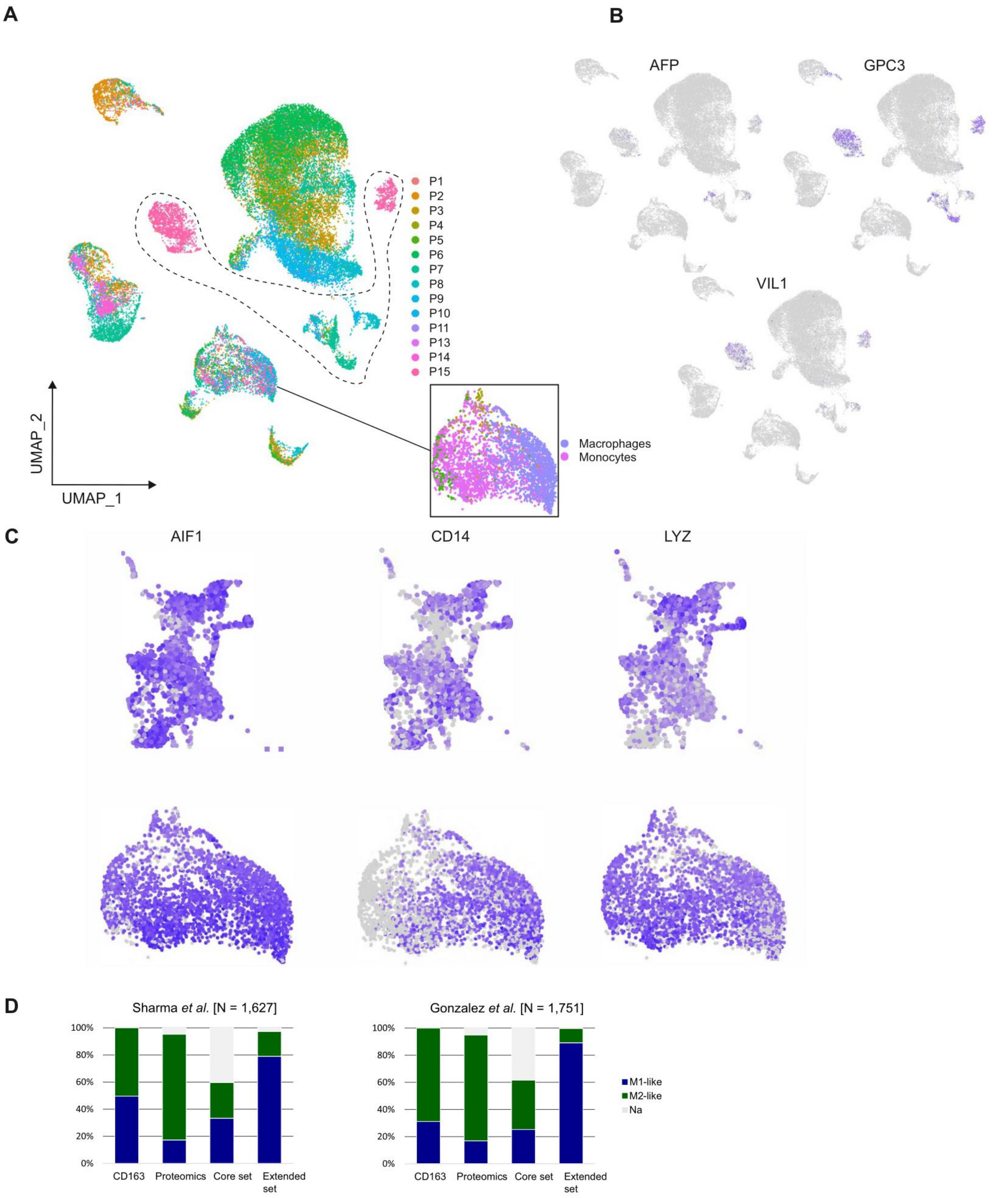

Figure S6. Macrophage annotation using proteomic signatures. (**A**) Two-dimensional visualization of 35'408 malignant (encircled) and non-malignant single cells based on marker gene expression. Colors represent the sample identities. A myeloid cluster (see also **Figure S4C**) is highlighted with colors representing SingleR annotations. (**B**) Feature plots of selected malignancy markers. (**C**) Feature plots of selected myeloid markers of the BrM (top row) and HCC (bottom row) myeloid cluster. (**D**) Barplots showing the percentages of annotated macrophages using different marker signatures among the BrM and HCC data sets.
